# Inositol Pyrophosphates and Their Unique Metabolic Complexity: Analysis by Gel Electrophoresis

**DOI:** 10.1371/journal.pone.0005580

**Published:** 2009-05-18

**Authors:** Oriana Losito, Zsolt Szijgyarto, Adam Cain Resnick, Adolfo Saiardi

**Affiliations:** 1 Medical Research Council (MRC) Cell Biology Unit and Laboratory for Molecular Cell Biology, Department of Cell and Developmental Biology, University College London, London, United States of America; 2 Division of Neurosurgery at the Children's Hospital of Philadelphia, Department of Neurosurgery, University of Pennsylvania School of Medicine, Philadelphia, Pennsylvania, United States of America; Bauer Research Foundation, United States of America

## Abstract

**Background:**

Inositol pyrophosphates are a recently characterized cell signalling molecules responsible for the pyrophosphorylation of protein substrates. Though likely involved in a wide range of cellular functions, the study of inositol pyrophosphates has suffered from a lack of readily available methods for their analysis.

**Principal Finding:**

We describe a novel, sensitive and rapid polyacrylamide gel electrophoresis (PAGE)-based method for the analysis of inositol pyrophosphates. Using 4′,6-diamidino-2-phenylindole (DAPI) and Toluidine Blue we demonstrate the unequivocal detection of various inositol pyrophosphate species.

**Conclusion:**

The use of the PAGE-based method reveals the likely underestimation of inositol pyrophosphates and their signalling contribution in cells when measured via traditional HPLC-based techniques. PAGE-based analyses also reveals the existence of a number of additional, previously uncharacterised pyrophosphorylated inositol reaction products, defining a more complex metabolism associated with the catalytically flexible kinase class responsible for the production of these highly energetic cell signalling molecules.

## Introduction

Myo-inositol is a structurally simple sugar that has been exploited by evolution to generate a multitude of phosphorylated molecules with key signalling roles [1]. Inositol pentakisphosphate (IP_5_) and phytic acid or inositol hexakisphosphate (IP_6_) are the two most abundant inositol polyphosphates in mammalian cells. They are also the precursors of inositol *pyro*phosphate molecules that contain one or more pyrophosphate bonds [2], [3]. Sequential phosphorylation of phytic acid gives rise to diphosphoinositol pentakisphosphate (IP_7_ or PP-IP_5_) and bisdiphosphoinositol tetrakisphosphate (IP_8_ or (PP)_2_-IP_4_). Likewise IP_5_ is the precursor of additional inositol pyrophosphates such diphosphoinositol tetrakisphosphate (PP-IP_4_) and other less characterized pyrophosphate-containing molecules that retain an unphosphorylated ring hydroxyl [2], [3]. Recently, an NMR study of pyrophosphate-containing inositols revealed that inositol pyrophosphate composition may indeed be more complex than previously realized, identifying the existence of a tri-phosphorylated species of “IP_8_” or PPP-IP_5_ [4].

Inositol pyrophosphates undergo rapid turnover in cells suggesting a potential signalling role for their metabolism [5]. Several studies have linked inositol pyrophosphates to disparate cellular functions from vesicular trafficking to telomere maintenance (for review see [2]). Their involvement in disease processes such as cancer and diabetes has also been suggested [6]–[9]. Given the higher free energy of hydrolysis possessed by the pyrophosphate moiety, soon after their initial discovery inositol pyrophosphates were suggested to participate in phosphotransferase reactions [10]. This hypothesis was verified [11]; recent further work has demonstrated that IP_7_ phosphorylates its substrates by donating its pyrophosphate β-phosphate moiety to pre-phosphorylated serine residues, generating a novel post-translational modification in the form of pyro-phosphorylated proteins [12].

Two distinct classes of evolutionarily conserved enzymes synthesize inositol pyrophosphates. The IP6Ks posses extraordinary catalytic flexibility, pyrophosphorylating IP_5_ and IP_6_ respectively to PP-IP_4_ and IP_7_ and subsequently using these enzymatic products as substrates for the generation of more complex molecules containing two or more additional pyrophosphate moieties or a tri-phosphate species [4], [13], [14]. Recently, a second class of pyrophosphate generating enzymes was identified in yeast [15]. Initially, Vip1 was described as a specific inositol hexakisphosphate kinase able to convert IP_6_ to IP_7_ [15]. Further work has suggested that this protein can also sequentially convert IP_6_ to IP_7_ and IP_8_. However, the kinetic parameters of the mammalian homolog (PPIP5K or IP7K) indicate that this enzyme is likely to physiologically convert IP_7_ to IP_8_ [16], [17] and thus might represent a previously identified IP7K activity [18].

The enzymatic conversion of IP_6_ to IP_7_ (IP_6_-Kinase reactions) or IP_7_ to IP_8_ (IP_7_-kinase reactions) are traditionally evaluated using a radiolabeled precursor such ^3^H-IP_6_ or ^32^P-IP_6_ which unfortunately are not commercially available. Mayr and colleagues have developed a chromatographic technique that utilizes a post-column modification of the phosphate groups to detect inositol polyphosphates using spectrophotometry [19]. However, this procedure is relatively insensitive and still requires separating the reaction products using sophisticated high-performance liquid chromatography (HPLC) apparatuses [19]. Here we describe a rapid, simple method for the analysis of highly phosphorylated inositol polyphosphates that takes advantage of the ease of polyacrylamide gel electrophoresis (PAGE) to resolve highly phosphorylated inositol polyphosphates combined with the use of 4′,6-diamidino-2-phenylindole (DAPI) to uniquely visualize inositol pyrophosphates. This simple and sensitive method allows for the reliable detection of nanomolar quantities of inositol pyrophosphates. Furthermore our application of PAGE to the investigation of the enzymatic activities of IP6K1 and Vip1 reveals an exceptionally robust inositol polyphosphate metabolism that has remained unidentified due to the lability of inositol pyrophosphates using HPLC-based protocols.

## Results

### Gel electrophoresis analyses of inositol polyphosphates

**Figure 1:**
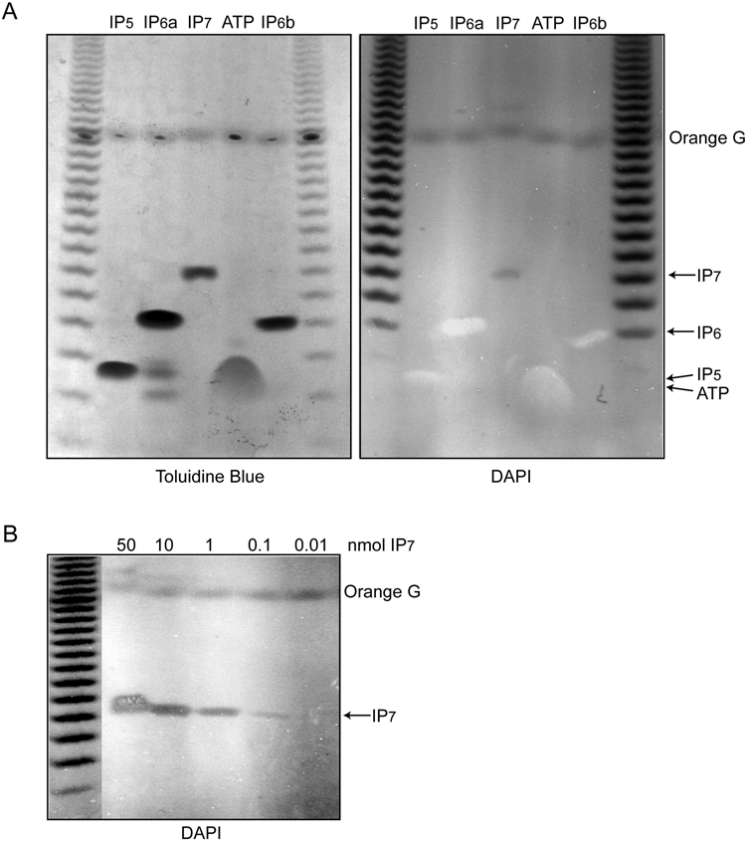
Separation and detection of inositol polyphosphates via PAGE. A) Analysis of 10 nmol of IP_5_ and IP_6_, 5 nmols of IP_7_, and 100 nmol ATP resolved on a 33.3% PAGE and stained with either Toluidine Blue (left panel) or DAPI (right panel). Under UV exposure DAPI staining displays positive staining for ATP, IP_5_, and IP_6_ while the inositol pyrophosphate, IP_7_, becomes negatively stained due to DAPI photobleaching. Similarly, the PolyP ladder used as an electrophoresis standard was also negatively stained. B) DAPI staining of serial dilutions of IP_7_ resolved on 33.3% polyacrylamide gel reveals remarkable sensitivity, detecting <100 pmols.

Traditional methods used for inositol pyrophosphate analysis utilize high-performance liquid chromatography using a strong anion exchange column (SAX-HPLC) [20]. Alternatively, inositol pyrophosphates can be analyzed using thin layer chromatography on polyethyleneimine cellulose (PEI-TLC) [13], [21]. However, PEI-TLC lacks significant resolving power and is therefore a little used technique. Both chromatographic techniques require the use of custom made radioactive precursors. While gel electrophoresis is also commonly used to separate and study small molecules, it has yet to be applied to inositol polyphosphates. To evaluate its applicability, we tested the use of PAGE and commonly used phosphate stains to resolve and detect inositol polyphosphates. We ran 5-10 nmols of IP_5_, IP_6_, IP_7_ and ATP on a 33.3% polyacrylamide gel and stained it with Toluidine Blue, a commonly used cationic metachromatic dye that binds phosphate groups (Figure 1A left). Phosphorylated inositols are easily resolved by gel electrophoresis with ATP migrating similarly to IP_5_ (Figure 1A left). Recently a report identified the staining of inorganic polyphosphate (PolyP) chains by DAPI [22], a bivalent commonly used dye that stains phosphate-rich compounds such as DNA. An identical gel run in parallel was stained with DAPI (Figure 1A right). Exposure of the gel to UV revealed that IP_5_, IP_6_ and ATP display positive staining while the inositol pyrophosphate, IP_7_, was negatively stained due to the photobleaching of DAPI. Similarly, the PolyP ladder used as an electrophoresis standard was also negatively stained, as reported [22]. It is noteworthy that the short PolyP forms are labeled by Toluidine Blue but remain unlabeled by DAPI, indicating that at least four polyphosphate moieties are required for negative staining by DAPI. Likely, the negative staining associated with IP_7_ is due to the highly negative charge of the fully phosphorylated inositol ring that, with the addition of the pyrophosphate moiety, results in DAPI photobleaching. We have found that DAPI and Toluidine Blue stains can be sequentially performed on the same gel; however the Toluidine Blue staining looses sensitivity.

We evaluated the detection limits for these new staining methods resolving serial dilutions of IP_7_ on polyacrylamide gels. Using Toluidine Blue, we were able to detect two nmols of IP_7_ (data not shown). DAPI staining was much more sensitive allowing for the detection of less than 100 pmols of IP_7_ (Figure 1B). Up to 100 nmols of the non-pyrophosphate containing IP_5_ or IP_6_ only stain positively by DAPI; however, quantities exceeding 200 nmols of IP_6_ become negatively stained by DAPI (data not shown). This staining distinction between inositol pyrophosphates and their precursors provides unprecedented ease in the evaluation of IP_6_-Kinase reactions.

### Analysis of IP_6_-Kinase reactions by PAGE

**Figure 2:**
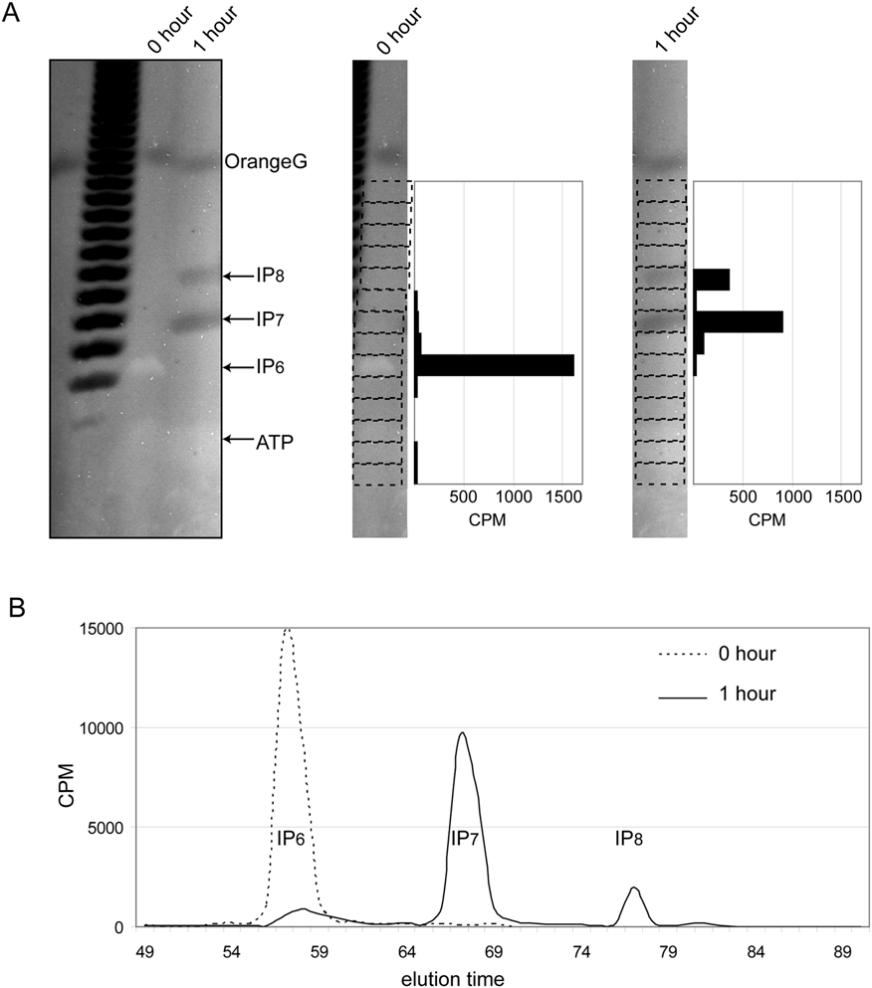
PAGE and SAX-HPLC analyses of IP_6_-Kinase reaction products. Kinase reactions using recombinant IP6K1 and 2 nmols of IP_6_ containing trace amounts of ^3^H-IP_6_ were run for zero or one hr at 37°C. (A) The reaction products were resolved by PAGE and subjected to DAPI staining. The gel was then cut as indicated and individual pieces subjected to scintillation counting. (B) The reaction products were analyzed by SAX-HPLC. The parallel analysis confirming that the DAPI stained bands on PAGE correspond to IP_7_ and IP_8_ as analyzed by SAX-HPLC. Comparison of PAGE and HPLC analyses reveals PAGE separation displays proportionally more efficient recovery of IP_8_ (see also Table 1).

We incubated 2 nmols of IP_6_ and trace amounts of ^3^H-IP_6_ with recombinant mouse His-IP6K1 and separated the reaction products by gel electrophoresis or by SAX-HPLC (Figure 2A,B). The reaction analysed by SAX-HPLC revealed the formation of radiolabeled IP_7_ and IP_8_ (Figure 2B). The equivalent enzymatic reaction separated by PAGE revealed the formation of two bands negatively labelled by DAPI (Figure 2A). These negatively stained bands migrated more slowly than IP_6_ and their migration distance relative to that of the PolyP marker indicated that they likely possess seven and eight phosphate groups, respectively. To verify that the negatively stained bands correspond to IP_7_ and IP_8_ generated by IP6K1, we cut 1 cm gel fragments and directly counted their radioactivity. This resulted in the recovery of only 20% of the input radioactivity (Figure 2A). However, dissolving the gel prior to scintillation counting resulted in 80–90% recovery of the input radioactivity (Table 1). In both cases the radioactivity initially contained in the positively stained bands of IP_6_ was redistributed in the negatively stained bands following IP6K1 enzymatic reactions, demonstrating that these bands correspond to the IP_7_ and IP_8_ reaction products (Figure 2A). These studies reveal that IP_8_ is actually a relatively more abundant reaction product when analysed by PAGE (Table 1), suggesting that SAX-HPLC analyses might selectively degrade IP_8_ (see below).

**Table 1:** Comparison of inositol pyrophosphate recovery between SAX-HPLC and PAGE technology.

IP_6_+IP6K1	SAX-HPLC (CPM)	%	PAGE (CPM)	%
IP_6_	1100(+/−345)	6.0	309 (+/−155)	1.7
IP_7_	14623(+/−976)	79.5	11078(+/−578)	63.3
IP_8_	2680 (+/−567)	14.6	6098(+/−897)	34.9

IP_5_+IP6K1	SAX-HPLC (CPM)	%	PAGE (CPM)	%
IP_5_	875(+/−295)	7.8	214(+/−112)	3.5
PP-IP_4_	2298(+/−456)	33.6	658(+/−301)	10.6
2P-IP_5_	2458(+/−561)	35.9	2678(+/−567)	43.2
3P-IP_5_	1086(+/−119)	15.9	1934(+/−427)	31.2
4P-IP_5_	127(+/−85)	1.9	456(+/−173)	7.4
5P-IP_5_	nd	-	252(+/−124)	4.0

Parallel kinase reactions containing IP6K1 and 2 nmols of IP_6_ or 2 nmol of IP_5_ and trace amounts of ^3^H-IP_6_ or ^3^H-IP_5_ were run for two hours at 37°C. One set of reaction products were analyzed by SAX-HPLC after incubating the samples for 20 minutes with percloric acid (1 M) on ice to precipitate the proteins. The second set of reactions were resolved by PAGE and subjected to DAPI staining, the bands corresponding to inositol polyphosphates were then cut and subjected to scintillation counting. The data (CPM) represent the averages+/−standard deviation of three independent experiments. The percent values represent the proportional ratio of the inositol polyphosphates species recovered.

**Figure 3:**
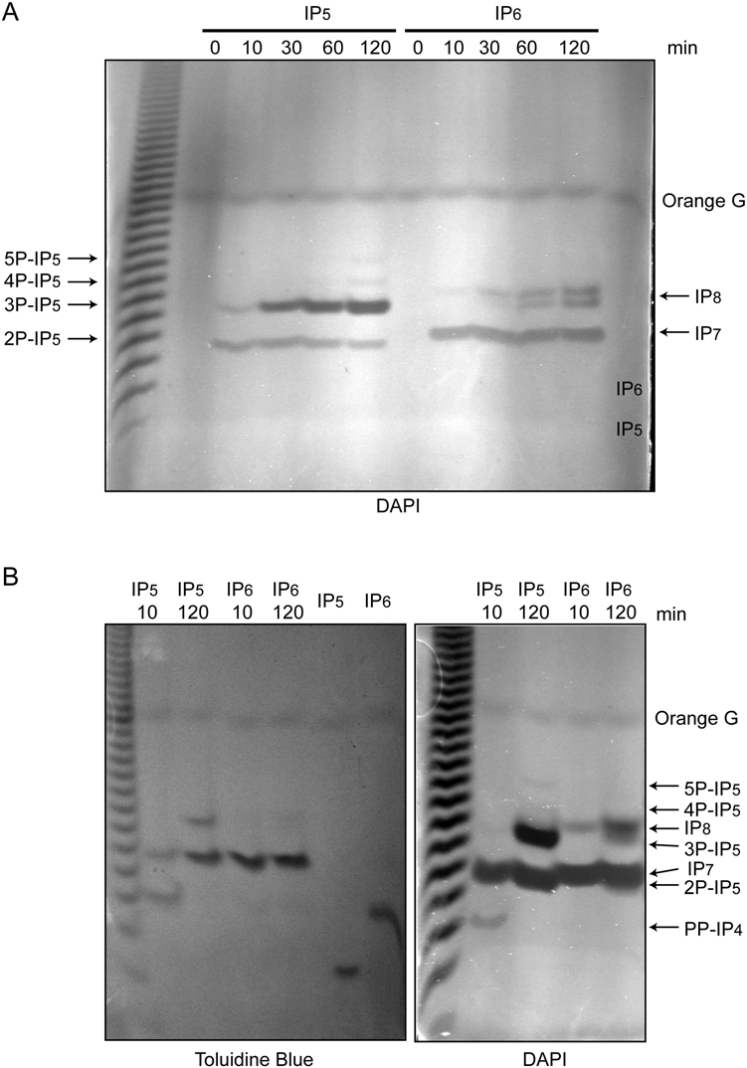
IP6K1 displays catalytic flexibility and multiple reaction products using both IP_5_ and IP_6_ as substrates. Kinase reactions were performed with recombinant IP6K1 using 2 nmols of IP_6_ or IP5 as substrate. (A) Time course analyses of reaction products using either IP_5_ or IP_6_ as substrate reveal multiple reaction products. (B) Increasing substrate concentrations to 10 nmols allows for the detection of PP-IP_4_ upon DAPI staining. Results demonstrate the differential ability of different inositol pyrophosphate to induce DAPI photobleaching.

We performed a time course experiment incubating IP_5_ and IP_6_ with recombinant His-IP6K1 (Figure 3A). Incubation for merely 10 min at 37°C already leads to the formation of two pyrophosphate species with both substrates. Using IP_5_ as a substrate, the time course reveals the formation of at least five different inositol pyrophosphate species (Figure 3A and B). Because the structure of IP_5_-derived inositol pyrophosphates may contain either a diphosphate or triphosphate species [4], we refer to the inositol pyrophosphates derived from IP_5_ as PP-IP_4_ to indicate bisdiphosphoinositol tetrakisphosphate and use 2P-IP_5_, 3P-IP_5_ etc. to indicate pyrophosphates derived from IP_5_ containing seven, eight, or more phosphate groups. The fast migrating band generated from IP_5_ migrates very close to that of IP_7_, suggesting that this inositol pyrophosphate species likely contains seven phosphate groups (2P-IP_5_) (Figure 3A) with PP-IP_4_ remaining undetected by DAPI staining. To verify this assumption we increased the amount of IP_5_ and IP_6_ used in our enzymatic reactions to 10 nmols and ran two parallel gels (Figure 3B). Following a 10 min reaction, Toluidine Blue detected the presence of PP-IP_4_ migrating as expected similarly to IP_6_. Furthermore the increase in substrate concentration allowed PP-IP_4_ to also be detected, albeit weakly, by DAPI (Figure 3B). Analysis of the relative intensities between the two staining methods revealed that different inositol pyrophosphate species have different DAPI photobleaching capacity. After a 10 min reaction, via Toluidine Blue PP-IP_4_ was more intensely stained than 2P-IP_5_; to the contrary DAPI displayed an opposite staining pattern (Figure 3B). Similarly, analysis of a 120 min reaction revealed that 3P-IP_5_ is far more capable of photobleaching DAPI than 2P-IP_5_. Parallel SAX-HPLC and PAGE analyses of His-IP6K1 reactions using trace amounts of ^3^H-IP_5_ and cold IP_5_ as substrate, revealed that the more highly phosphorylated forms of inositol pyrophosphates are dramatically underrepresented by traditional HPLC-based chromatographic techniques (Table 1). These studies indicate, as in the case of IP_8_, that the SAX-HPLC method results in the selective degradation of some inositol pyrophosphate species (Table 1) (see below).

Time course analyses of IP6K1 reactions using IP_6_ as a substrate revealed the production of IP_7_ and two additional, more phosphorylated species (Figure 3A); due to their close migration distance, it is likely that these bands represent two different forms of an inositol pyrophosphate containing eight phosphate groups “IP8”. One likely represents the recently described triphosphate species PPP-IP_5_ [4]. The analyses of IP6K1 reactions run for longer times, such as overnight, using IP_5_, IP_6_ and IP_7_ (isomer 5PP-IP_5_ synthesized by IP6K1) as substrates surprisingly revealed the production of novel, highly phosphorylated species. Using IP_5_ as a substrate we observed the formation of large amounts of 4P-IP_5_ and 5P-IP_5_ products (Supporting Figure S1). Using IP_6_ and IP_7_ as substrates revealed the production of even more phosphorylated species; given their relative migration distance, it is likely that bands generated using IP_6_ and IP_7_ as substrates represent inositol pyrophosphates containing 12 or even 13 phosphate groups “IP_13_” (Supporting Figure S1). PAGE analysis reveals the ability of IP6K1 to synthesize *in vitro* a far greater number of inositol pyrophosphate species than previously appreciated. The challenging aspect of future research focuses on the *in vivo* identification of such higher phosphorylated inositol pyrophosphate species.

PAGE analysis allows for the first time for the evaluation of all six possible IP_5_ isomers as substrates for IP6K1. Incubation of the purified enzyme with each of the IP_5_ isomers for two hours at 37°C revealed the ability of IP6K1 to metabolize five of the isomers with only I(1,2,3,5,6)P_5_ failing to convert to 2P-IP_5_. However, only the most abundant and biologically relevant I(1,3,4,5,6)P_5_ isomer is further phosphorylated to generate more complex inositol pyrophosphates (Supporting Figure S2). These data further demonstrates the catalytic flexibility of the IPK family of enzymes [23].

### Analysis of Vip1 kinase activity by PAGE

**Figure 4:**
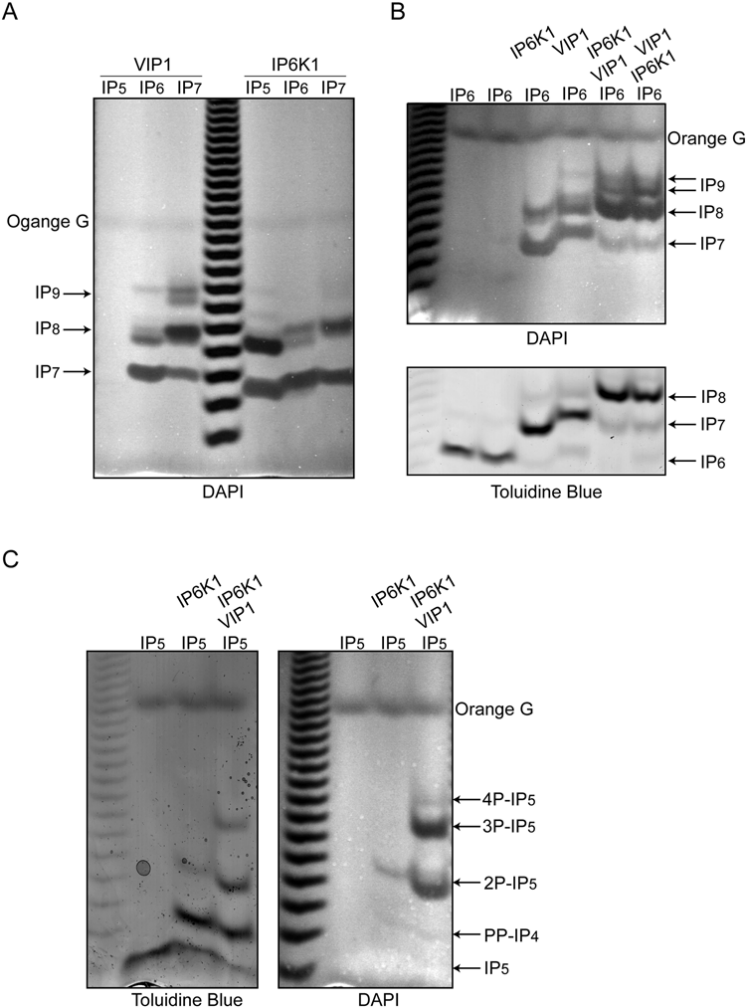
Comparison of Vip1 and IP6K1 inositol pyrophosphorylation activities. Kinase reactions were performed with recombinant IP6K1 and Vip1 using 2 nmols of IP_5_ or IP_6_ and IP_7_ as substrate for two hrs at 37°C. A) Vip1 and IP6K1 possess differing substrate specificities and capacities for the sequential phosphoryaltion of insotiol pyrophosphates. B) Vip1 and IP6K can function sequentially to generate IP_8_ and IP_9_ using IP_6_ as a substrate. Toulidine Blue staining (bottom) was performed sequentially following DAPI staining. C) Vip1 can function to sequentially phosphorylate IP6K1 reaction products generated using IP_5_ as the initial substrate. Toulidine Blue staining (left) was performed sequentially following DAPI staining.

Recently, a new class of pyrophosphorylating enzymes was discovered consisting of kinases which are also capable of sequentially converting IP_6_ to IP_7_ to IP_8_. The yeast protein, Vip1, was originally described as an IP_6_-Kinase responsible of converting IP_6_ to IP_7_ [15]. However, the mammalian homolog (PP-IP5K) has kinetic characteristics suggesting it may represent a physiological IP_7_-Kinase (IP7K) [16], [17]. Initially, we performed a time course experiment incubating IP_6_ with recombinant yeast Vip1 (Supporting Figure S3). Incubation for merely 5 min at 37°C already leads to the formation of two pyrophosphate species with the formation of at least four species after two hours of incubation (Supporting Figure S3). We then decided to examine the enzymatic activity of Vip1 via gel electrophoresis in three parallel reactions using IP_5_, IP_6_, or IP_7_ as a substrate. Incubation of recombinant Vip1 with the three different substrates for two hours at 37°C revealed the inability of this enzyme to metabolize IP_5_, as previously reported [16] (Figure 4A). When IP_6_ is used as a substrate, it is rapidly converted to IP_7_, IP_8_, and a further phosphorylated form containing nine phosphate groups. When IP_7_ (isomer 5PP-IP_5_ synthesized by IP6K1) is used as a substrate, an IP_8_ species is formed that migrates slightly slower than that generated by the sequential phosphorylation of IP_6_ to IP_8_, suggesting a different isomer. Because Vip1 was described to pyrophosphorylate the racemic 1 and 3 ring positions [24], conceivably the IP_8_ generated from IP_6_ represents the (1,3)PP-IP_5_ isomer while the ((1 or 3),5)PP-IP_5_ is the isomer generated using 5PP-IP_5_ as a substrate. Using 5PP-IP_5_ as a substrate, two further phosphorylated inositol species were detected (Figure 4A) with their relative migration distance suggesting they represent two different IP_9_ isoforms. One of the IP_9_ species co-migrates with IP_9_ generated using IP_6_ as a substrate (Figure 4A). We tested if the inositol pyrophosphate products generated by IP6K1 and Vip1 can be reciprocally exchanged as substrates (Figure 4B). The parallel analysis of IP6K1 and Vip1 reactions using IP_6_ as a substrate revealed slightly different IP_7_ and IP_8_ migration rates (Figure 4A and B), indicating that the two enzymes generate different inositol pyrophosphate isomers as recently demonstrated [4], [24]. However, adding Vip1 to the IP6K1 reaction or adding IP6K1 to Vip1 reactions generates similar inositol pyrophosphate products with the abundant formation of IP_8_ and a robust synthesis of more phosphorylated IP_9_ inositol pyrophosphate species (Figure 4B). Similarly, we tested Vip1 activity towards the inositol pyrophosphates generated by IP6K1 using IP_5_ as a substrate (Figure 4C). IP_5_ was first incubated with IP6K1 and Vip1 was subsequently added to the assay. This reaction resulted in the vigorous production of 2P-IP_5_ and 3P-IP_5_ species indicating that PP-IP_4_ and perhaps other inositol pyrophosphates generated from IP_5_ by IP6K1 are genuine enzymatic substrates of Vip1. These conclusions have been confirmed *in vivo* by our analysis of the inositol polyphosphate profile of the *ipk1Δvip1Δ* double mutant than shows a massive accumulation of PP-IP_4_ (Supporting Figure S4) suggesting that the Vip1 catalytic activity is more promiscuous than previously described.

### Inositol pyrophosphates are sensitive to acidic degradation

**Figure 5:**
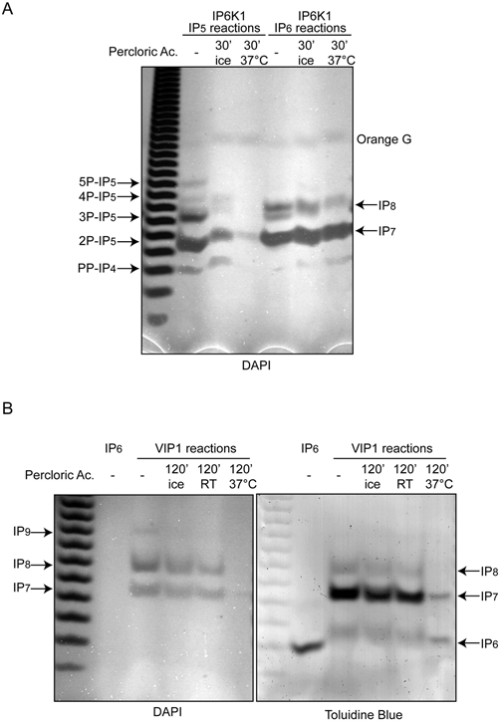
Conditions simulating traditional acidic extraction and chromatographic analysis result in the degradation of various inositol pyrophosphate species. Kinase reactions were performed with recombinant IP6K1 and Vip1 using 5 nmols of IP_5_ or IP_6_ for two hrs at 37°C, stopped by adding EDTA, and incubated with percloric acid at the indicated temperature and time. The neutralized reactions were then subjected to PAGE analysis. A) IP6K1 reaction products, especially those derived using IP_5_ as a substrate, display pronounced sensitivity to acidic conditions. B) Likewise, Vip1 reaction products are also degraded and are likely under-represented in traditional SAX-HPLC analysis. Incubation for two hrs at 37°C reveals the degradation of IP_8_ as well as IP_7_ to their precursor IP_6_. Toulidine Blue staining (right) was performed sequentially following DAPI staining.

The parallel analyses by SAX-HPLC and PAGE of IP6K1 enzymatic products revealed a consistent under-representation of highly phosphorylated inositol pyrophosphate species in the chromatographic studies (Table 1). One of the main differences between SAX-HPLC and PAGE analyses is the requirement in the former for acidic buffer (pH 3.8) running conditions and the deproteination of the sample, typically using high concentrations of perchloric acid. In contrast, for PAGE analysis the samples are unprocessed and loaded directly into the gel at pH 8.0. To test if acidic conditions may be responsible for inositol pyrophosphate degradation, we incubated IP6K1 reactions for 30 min at room temperature or at 37°C in 1 M percloric acid before neutralizing with potassium carbonate (Figure 5A). Surprisingly, we observed a dramatic degradation of IP_5_ derived inositol pyrophosphates and a substantial degradation of IP_8_, with the complete disappearance of the faster migrating species (Figure 5A). Similarly, Vip1-generated inositol pyrophosphates were also sensitive to acidic conditions. We observed the disappearance of the IP_9_ band and substantially decreased DAPI staining when the sample was incubated on ice (Figure 5B). Further degradation was observed when the sample was incubated at room temperature while at 37°C almost no inositol pyrophosphates were observed with the resultant generation of IP_6_ detected by Toluidine Blue staining (Figure 5B).

SAX-HPLC sample preparation usually consists of incubating the reaction mix or cells with percloric acid (1–2 M) for 20–30 minutes on ice before neutralization [20], [25]. Furthermore, the column separation itself occurs at pH 4.0 or lowers for 1–2 hrs at RT. The acidic conditions used in our experiments therefore simulate the conditions inositol pyrophosphates are typically subjected to before and during SAX-HPLC analysis. Our PAGE analyses support what is likely to be the routine under-representation of the true quantity and metabolic complexity of inositol pyrophosphates and their potential signalling roles in biology.

## Discussion

Inositol pyrophosphates are attracting increased attention for their possible role as a signalling molecule and have been linked to a wide range of biological functions, including vesicular trafficking, apoptosis, DNA repair, telomere maintenance, and stress responses [2]. Similarly, important human diseases such cancer and diabetes appear to be under inositol pyrophosphate control [6]–[9]. However, significantly more research is necessary for the elucidation of the full spectrum of physiological mechanisms controlled by this class of signalling molecules. Unfortunately, the current state of the art experimental techniques used to analyze inositol pyrophosphate metabolism require sophisticated apparatuses such an HPLC machine as well as the ability to synthesize and purify radiolabeled precursors. In contrast, PAGE separation simply requires a universally available gel electrophoresis apparatus. Furthermore, we've identified DAPI as a reliable and very sensitive stain that allows for the detection of the pyrophosphate moiety. The combination of PAGE and DAPI staining represents a rapid, easy and widely available method to the evaluation of inositol pyrophosphates. Here, we used this method to study IP6K1 and Vip1 enzymatic reactions, revealing the existence of a number of additional, previously uncharacterised pyrophosphorylated inositol species. More importantly, parallel analyses comparing SAX-HPLC- and PAGE-based methods reveal a significant underestimation of the quantity and composition of inositol pyrophosphate metabolism. Inositol pyrophosphates are typically subjected to acidic conditions before and during SAX-HPLC analysis. The study of these treatments using PAGE revealed the degrading effects of such actions.

The measurement of inositol pyrophosphates extracted from cells would represent the next step forward in the application of the new PAGE technology. Unfortunately, our current efforts have failed to optimize a successful protocol (Supporting Figure S5). Using either mammalian or yeast cells, PAGE analysis of acidic extraction from cells revealed the co-purification of molecules that migrate similarly to inositol pyrophosphates. Perchloric acid treatment, commonly used for inositol polyphosphate extraction of yeast cells [20], mainly extracts inorganic polyphosphates that are very abundant in this organism [26] and are negatively stained by DAPI [22]. The co-purification of PolyPs obscures the inositol pyrophosphates present in the cell extract, making them unidentifiable (Supporting Figure S5). We are currently developing enzymatic strategies to remove co-purifying molecules to allow for the measurement of inositol pyrophosphates extracted from cells. Furthermore, to fully appreciate the complexity of inositol pyrophosphates' cellular metabolism new extraction methods need to be developed. Routinely, extracting inositol pyrophosphates from cells or tissues requires the use of strongly acidic conditions. We demonstrated that this treatment might degrade inositol pyrophosphates, especially the most phosphorylated species. Consequently only the development of new, milder extraction techniques will allow for the full appreciation of the metabolic complexity of the inositol pyrophosphates.

Our identification of DAPI staining's ability to differentiate between inositol pyrophosphates and their precursor provides a useful tool for the rapid analysis of *in vitro* IP_5-6-7-8_-Kinase reactions. The evident degradation of inositol pyrophosphates under the acidic conditions traditionally used for their analysis suggests alternate methods must be developed for their *in vivo* evaluation as well. DAPI staining may indeed provide such a technique. DAPI is widely used as a DNA stain for fluorescence microscopy, emitting in the blue spectrum at 456 nm. DAPI has also been used to label cellular pools of PolyP, where it emits in the yellow spectrum at 540 nm [27]. We are currently evaluating the fluorescence emission spectrum of DAPI and related molecules when bound to various inositol pyrophosphates. Development of such a labelling method may ultimately permit for the evaluation of the dynamic metabolism of inositol pyrophosphates in intact cells.

Finally, the availability of a rapid method for analyzing the IP6Ks/Vip1s reactions allows for the identification of small molecule inhibitors or enhancers using a small chemical compound library. Conversion of IP_6_ to higher inositol pyrophosphates can be easily analysed on small 10×6 cm gels (data not shown) that can be prepared, run, and stained in less than two hours, allowing for the simultaneous analysis of 100 s of reactions. Using traditional HPLC-based assays such analyses would be entirely impractical. The potential therapeutic potential of such compounds is supported by the recent identification of the critical role inositol pyrophosphates play in insulin secretion and oncongenic processes [6]–[9]. Though the inositol pyrophosphate field appears to be more complex than previously described, our identification of a PAGE-based analytic method will serve to increase both access to and the ease with which we can study these highly energetic cell signalling molecules.

## Methods

### Reagents

Polyacrylamide mix, TEMED, ammonium persulfate, the gel solubilizer Solusol and the scintillation cocktail Solucint-O were acquired from National Diagnostic; all others reagents were purchased from the Sigma-Aldrich Company. A second source of phytic acid was purchased from Calbiochem. Tritium [^3^H]-IP_5_ and [^3^H]-IP_6_ were purified from *ipk1*Δ and *kcs1*Δ mutant yeast respectively, as previously described [20], [25]. IP_7_ was synthesized with IP6K1 and purified as previously described [11], [20]. The plasmids expressing His-IP6K1 (mouse), and GST-Vip1 (yeast, kinase domain) and the procedures for purifying the respective recombinant enzymes were previously described [12], [28].

### Inositol polyphosphates kinase reactions

The reaction mix contained: 2 µl 5× Buffer (150 mM Hepes 6.8; 250 mM NaCl; 30 mM MgSO_4_; 5 mM DTT; 5 mM NaF); 0.5 µl phosphocreatine (200 mM); 0.5 µl creatine phosphokinase (800 U/µl) ; 0.5 µl ATP-Mg (10 mM) ; 2–10 nmol IP_5_/IP_6_/IP_7_; and 5 to 30 ng of the appropriate enzyme. Trace amounts of [^3^H]-IP_5_ or [^3^H]-IP_6_ (∼20,000 CPMs) was added when indicated. The reactions were incubated at 37°C for the indicated times. Reactions were then stopped by the addition of 2 µl EDTA (100 mM) and placed on ice. The samples were then run on a polyacrylamide gel, frozen at −20°C, or processed for SAX-HPLC analysis as previously described [25]. Briefly, 50 µl of 1 M perchloric acid was added to the samples followed by the addition of 25–30 µl of 1 M potassium carbonate containing 3 mM EDTA to neutralize the mixture.

### Fractionation of inositol polyphosphates by PAGE

Inositol polyphosphates were resolved using 24×16×0.1 cm gel using 33.3% polyacrylamide gel in TBE (31.7 ml 40% Acr/Bis (19∶1); 3.8 ml 10× TBE; 2.2 ml H_2_0; 270 µl 10% APS; 30 µl TEMED). Gels were pre-run for 20 minutes at 300 volts. Then 5–10 µl of 6× Dye (10 mM TrisHCl pH 7.0; 1 mM EDTA; 30% glycerol; 0.1% Orange G) was added to each sample prior to loading onto gels. Gels were run at 300–400 volts overnight at 4°C until the Orange G dye front reached 10 cm from the gel's bottom. To analyse in gel radioactivity distribution, serial, one cm gel fragments were cut after DAPI staining over a UV transilluminator. Gel fragments were incubated overnight with 1–2 ml of the gel solubilizer Solusol; 15–20 ml of Solucint-O cocktail was added and radioactivity was assessed with a β-counter.

### Toluidine Blue staining

Gels were gently agitated for 30 min at room temperature in a filtered staining solution (20% methanol; 2% glycerol; 0.05% Toulidine Blue), then destained for two hrs with several changes of the same solution without dye. Pictures were taken after exposing the gel on a white light transilluminator.

### DAPI staining

Gels were gently agitated for 30 min at room temperature in staining solution (20% methanol; 2% glycerol; 20 mM Tris base; 2 µg/ml DAPI); gels were destained for 45 min in the same solution without DAPI and exposed at 365 nm over a UV transilluminator for 2–10 min to induce photobleaching, after which photographs were taken.

## Supporting Information

Figure S1 IP6K1 displays the ability to synthesize IP13 in vitro.
(0.24 MB PDF)

Figure S2 IP6K1 displays a wide range of IP5 isomeric substrate specificities.
(0.20 MB PDF)

Figure S3 Time course analyses of VIP1 reaction products.
(0.20 MB PDF)

Figure S4 Inositol polyphosphate profile of ipk1Δ and ipk1Δvip1Δ.
(0.12 MB PDF)

Figure S5 PAGE analysis of inositol polyphosphates extracted from wild type and kcs1Δ yeast.
(0.16 MB PDF)
