# Supplementary material for: Inositol Pyrophosphates and Their Unique Metabolic Complexity: Analysis by Gel Electrophoresis
Source: PLoS One. 2009 May 18;4(5):e5580. doi: 10.1371/journal.pone.0005580 (PMC2680042; doi:10.1371/journal.pone.0005580)
Supplement: Figure S1 — IP6K1 displays the ability to synthesize IP13 in vitro. (0.24 MB PDF) [file pone.0005580.s001.pdf]

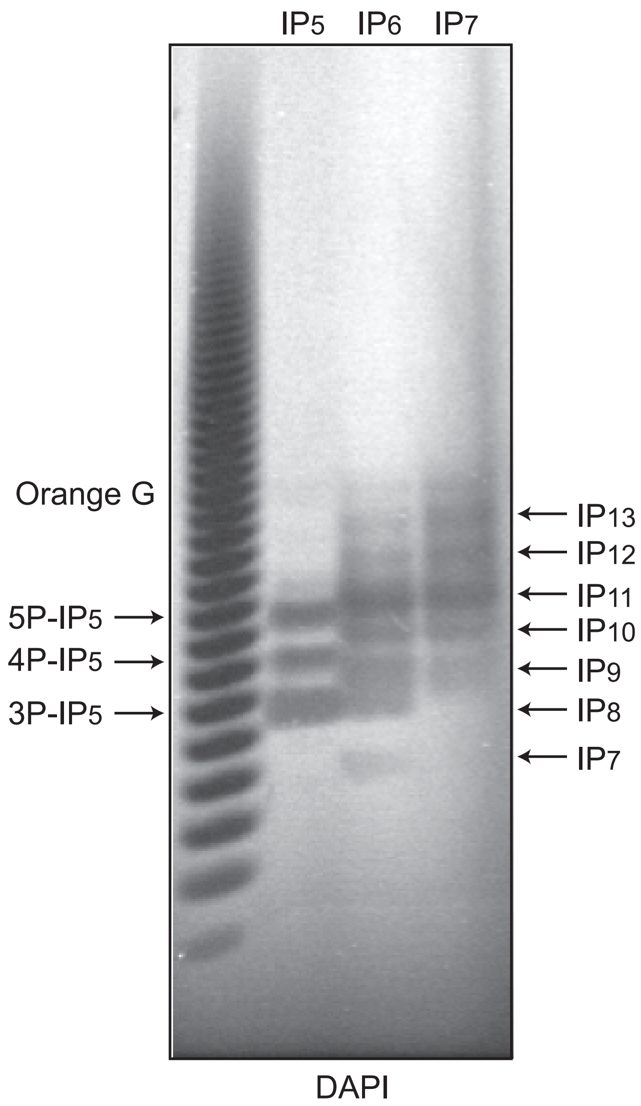

Supporting Figure S1. IP6K1 displays the ability to synthesize IP_{13} in vitro.
Recombinant IP6K1 was incubated overnight with 2 nmols of IP_5, IP_6 or IP_7 at 37°C.
The reactions were resolved on a 33.3% polyacrylamide gel and visualized by DAPI staining.
