# Supplementary material for: Inositol Pyrophosphates and Their Unique Metabolic Complexity: Analysis by Gel Electrophoresis
Source: PLoS One. 2009 May 18;4(5):e5580. doi: 10.1371/journal.pone.0005580 (PMC2680042; doi:10.1371/journal.pone.0005580)
Supplement: Figure S2 — IP6K1 displays a wide range of IP5 isomeric substrate specificities. (0.20 MB PDF) [file pone.0005580.s002.pdf]

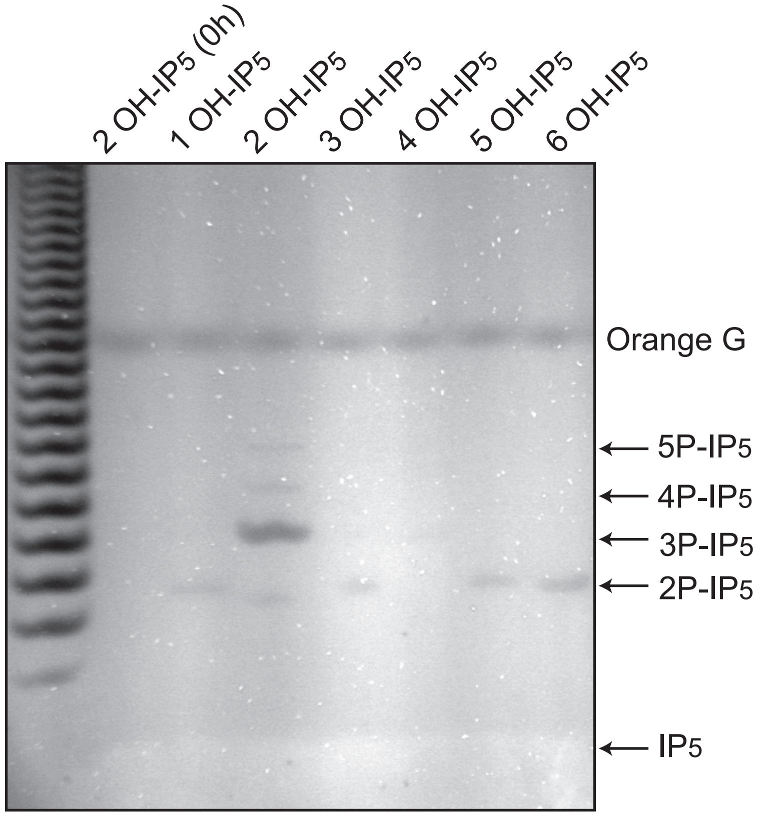

Supporting Figure S2. IP6K1 displays a wide range of IP_5 isomeric substrate specificities. Recombinant IP6K1 was incubated with 2 nmols of each of the six isomers of IP_5 for 2 hrs at 37°C, resolved on a 33.3% polyacrylamide gel and visualized by DAPI staining.
