# Supplementary material for: Inositol Pyrophosphates and Their Unique Metabolic Complexity: Analysis by Gel Electrophoresis
Source: PLoS One. 2009 May 18;4(5):e5580. doi: 10.1371/journal.pone.0005580 (PMC2680042; doi:10.1371/journal.pone.0005580)
Supplement: Figure S3 — Time course analyses of VIP1 reaction products. (0.20 MB PDF) [file pone.0005580.s003.pdf]

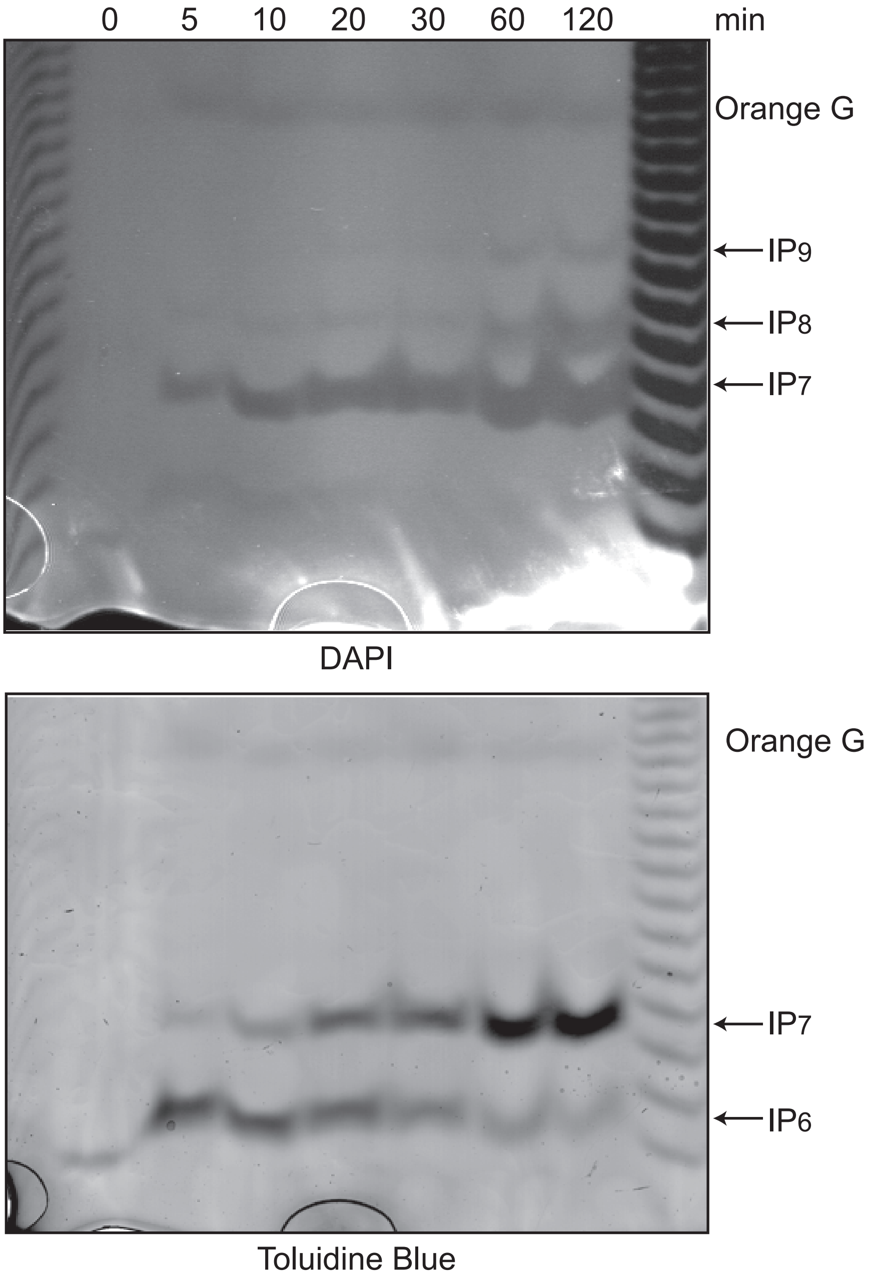


Supporting Figure S3. Time course analyses of VIP1 reaction products. Kinase reactions were performed with recombinant VIP1 using 2 nmols of $IP_6$ as substrate incubated at 37°C for various times, resolved on a 33.3% polyacrylamide gel, and visualized first by DAPI staining and then by Toluidine Blue.
