# Supplementary material for: Inositol Pyrophosphates and Their Unique Metabolic Complexity: Analysis by Gel Electrophoresis
Source: PLoS One. 2009 May 18;4(5):e5580. doi: 10.1371/journal.pone.0005580 (PMC2680042; doi:10.1371/journal.pone.0005580)
Supplement: Figure S4 — Inositol polyphosphate profile of ipk1Δ and ipk1Δvip1Δ. (0.12 MB PDF) [file pone.0005580.s004.pdf]

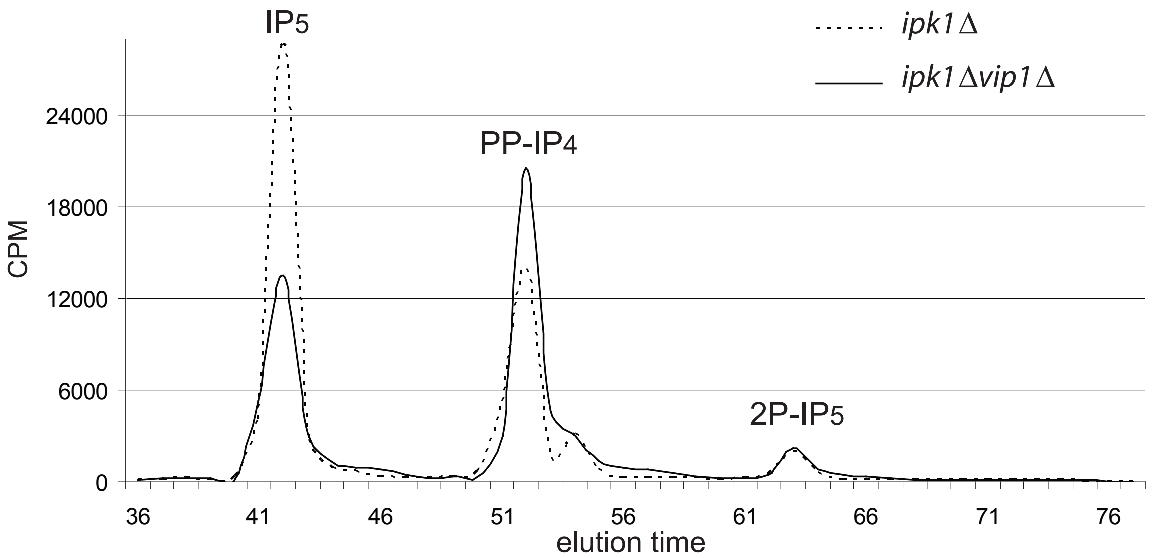

Supporting Figure S4. Insositol polyphosphate profile of *ipk1*Δ and *ipk1*Δvip1Δ. Yeast were labeled overnight with [3H]-inositol and inositol polyphosphates were extracted and analysed by SAX-HPLC [1]. The analysis of *ipk1*Δ and *ipk1*Δ*vip1*Δ yeast revealed the accumulation of PP-IP_4 in the double mutant cells. The ratio PP-IP_4 to IP_5 in the of *ipk1*Δ and *ipk1*Δ*vip1*Δ yeast are 61.1+/-5.8% and 137.7+/-14.9% respectively. The inositol polyphosphate profile shown is representative of three indipendent experiments.

Reference

1. Azevedo C. and Saiardi A. (2006) Extraction and analysis of soluble inositol polyphosphates from yeast. Nat Protoc. 1: 2416-22
