# Supplementary material for: Inositol Pyrophosphates and Their Unique Metabolic Complexity: Analysis by Gel Electrophoresis
Source: PLoS One. 2009 May 18;4(5):e5580. doi: 10.1371/journal.pone.0005580 (PMC2680042; doi:10.1371/journal.pone.0005580)
Supplement: Figure S5 — PAGE analysis of inositol polyphosphates extracted from wild type and kcs1Δ yeast. (0.16 MB PDF) [file pone.0005580.s005.pdf]

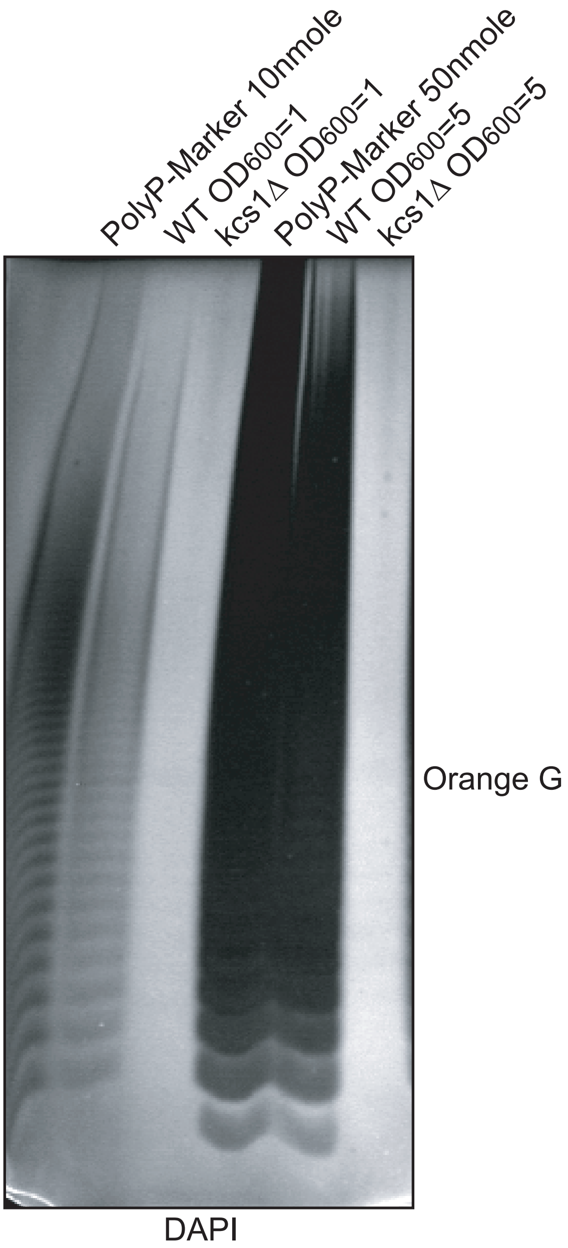

Supporting Figure S5. PAGE analysis of inositol polyphosphates extracted from wild type and kcs1Δ yeast. Inositol polyphosphates were extracted as previously described [1] from one or five OD600 of early logarithmic growth cultures. The samples were resolved on 33.3% polyacrylamide gel and visualized by DAPI staining revealing the presence of a ladder of negatively stained bands in wild type (WT) yeast. This intense laddering is typical of inorganic polyphosphate (PolyP), a phosphate polymer that is abundant in yeast [2]. The co-purification of PolyP obscures the inositol pyrophosphates making them undetectable. PolyP synthesis is dramatically reduced in kcs1Δ yeast [3]. Our analysis confirms these data revealing the absence of any detectable PolyP [3] or inositol pyrophosphates [4] in kcs1Δ cells.

References

1. Azevedo C. and Saiardi A. (2006) Extraction and analysis of soluble inositol polyphosphates from yeast. Nat Protoc. 1: 2416-22
2. Kornberg A, Rao NN, Ault-Riché D. (1999) Inorganic polyphosphate: a molecule of many functions. Annu Rev Biochem. 68:89-125.
3. Auesukaree C, Tochio H, Shirakawa M, Kaneko Y, Harashima S. (2005) Plc1p, Arg82p, and Kcs1p, enzymes involved in inositol pyrophosphate synthesis, are essential for phosphate regulation and polyphosphate accumulation in Saccharomyces cerevisiae. J Biol Chem. 280:25127-33.
4. Dubois E, Scherens B, Vierendeels F, Ho MM, Messenguy F, Shears SB. (2002) In Saccharomyces cerevisiae, the inositol polyphosphate kinase activity of Kcs1p is required for resistance to salt stress, cell wall integrity, and vacuolar morphogenesis. J Biol Chem. 277:23755-63
